# Manual Dexterity and Intralimb Coordination Assessment to Distinguish Different Levels of Impairment in Boccia Players with Cerebral Palsy

**DOI:** 10.3389/fneur.2017.00582

**Published:** 2017-11-10

**Authors:** Alba Roldan, Rafael Sabido, David Barbado, Carla Caballero, Raúl Reina

**Affiliations:** ^1^Miguel Hernández University, Sport Research Center, Elche, Alicante, Spain; ^2^Rutgers University, The State University of New Jersey, New Brunswick, NJ, United States

**Keywords:** paralympic, cerebral palsy, neurological impairment, para-sport, Box and Block, tapping test

## Abstract

**Background:**

Boccia is a paralympic sport played by athletes with severe neurological impairments affecting all four limbs. Impaired manual dexterity (MD) and intralimb coordination (ILC) may limit individuals’ ability to perform certain activities such as grasping, releasing, or manipulating objects, which are essential tasks for daily life or to participate in para sports such as boccia. However, there are currently no specific instruments available to assess hand–arm coordination in boccia players with severe cerebral palsy (CP).

**Purpose:**

To design new sport-specific coordination tests to assess impaired MD and ILC in boccia players; afterward, quantify to what extent their coordination is impaired compared to a control group (CG) without neurological impairments.

**Methods:**

Seventy-three recreational boccia players with severe CP (BC1: age = 34.01 ± 16.43 years; BC2: age = 33.97 ± 14.29 years), and 19 healthy adults (age = 27.89 ± 7.08 years) completed the test battery. The Box and Block test (BBT) and Box and Ball test (BBLT) were used to assess MD and four tapping tests to assess upper ILC.

**Results:**

Both MD tests were able to discriminate between sport classes. Boccia players obtained better scores in the BBLT in comparison to the BBT, showing that the BBLT had more appropriate testing features. On the other hand, only one of the ILC tests was able to discriminate between sport classes, displaying the highest practical significance (*d* = −1.12). Participants with CP scored significantly worse in all the coordination tests compared to the CG.

**Conclusion:**

Using sport-specific equipment facilitated grasp function during the MD assessment. Regarding the ILC, the type of movement (continuous vs. discrete) seems to be more relevant for classification than the movement direction (vertical vs. horizontal) or the presence of a ball.

## Introduction

Boccia is a strategic game that demands high coordination and control of movement to achieve accuracy (1). Boccia promotes sport practice for people with permanent and severe neurological impairments [e.g., cerebral palsy (CP)] and other severe locomotor impairments affecting all four limbs (2), grouping para-athletes in five sport classes (from BC1 to BC5). Specifically, BC1 hosts players with CP diagnosed with spastic quadriplegia or athetosis, or those with severe ataxia, whereas BC2 hosts CP players diagnosed with spastic quadriplegia or with athetosis/ataxia (2). Players belonging to these sport classes tend to show high coordination problems (3).

To achieve a fair competition, sport classification aims to cluster athletes into sport classes in which the least impaired athletes still have the best chances to win (4). However, a major limitation in some paralympic sports is the lack of evidence-based assessment methods to assess the degree of impairment (i.e., impaired coordination in Boccia) and its effect on sport proficiency (5). Therefore, transparent and consistent classification methods are necessary.

The Boccia Classification Rulebook (2) indicates that coordination assessment should focus on manual dexterity (MD) and intralimb coordination (ILC). MD is defined as the ability to make precise hand and finger movements to grasp and manipulate objects (6). MD is widely assessed in people with CP, as they usually demonstrate difficulties performing manual activities due to hand movement abnormalities, such as thumb adduction or flexion with limited wrist extension, causing activity limitation when performing activities of daily living (7). On the other hand, ILC is defined as the coupling of two or more joints in the same limb (8). The ability to perform basic skills such as grasping, releasing, and following through with a ball or being able to achieve a good throwing position (e.g., elbow flexion-extension and shoulder abduction) seems relevant to succeed in boccia. Thus, all these actions must be taken into consideration and assessed during classification (2).

Coordination in boccia is currently assessed through non-standardized methods such as the finger-to-nose test [included in the Scale for the Assessment and Rating of Ataxia (SARA)] (9), which quantifies the degree of impaired coordination through a ratio scale based on the observation of the tremor or inaccuracy. More specifically, MD is usually assessed by asking the player to hold a ball while the classifier tries to remove it from his or her hand or by asking them to release the ball after a verbal command. On the other hand, ILC is usually assessed by asking the player to throw the ball to different areas of the boccia court, evaluating the player’s accuracy and/or force control, observing the preparation, release, and follow-through. These evaluation methods are highly dependent on the rater experience, so more objective coordination assessments should be implemented considering other quantitative outcomes like time or accuracy (10). For example, a common clinical test used to assess MD in individuals with CP is the Box and Block Test (BBT), which is considered as a gold standard to evaluate grasping, holding, and releasing (11). This test has simple execution rules, and it has been validated for people with neurological impairments such as stroke or CP (12, 13), demonstrating good reliability (14, 15). Although BBT requires specific skills similar to boccia (i.e., grasping and releasing motions), the development of a coordination test that involves specific sports equipment may be relevant (e.g., boccia balls), as this study does.

Regarding the assessment of ILC, hand–finger tapping tests are used in clinical settings to assess upper-limb muscle control (16), even in individuals with mild-to-moderate CP (17). This type of tests, which are mainly based on the Fitts’ Law postulates (18), requires participants to perform discrete or reciprocal finger–hand motions on a surface as quickly and accurately as possible in a specific period of time or to perform a certain number of strikes of such motions. Recently, such tests have successfully been applied in the para-sports context for classification purposes, including wheelchair racing, running, jumping, and throwing events (19) or even as a potential tool to identify intentional misrepresentation in para-athletes (20). However, these studies have been carried out only with individuals without disabilities, biasing its application in individuals with CP.

Based on the literature limitations, the implementation of sport-specific coordination tests in para-athletes to assess MD and ILC is pertinent, especially in those with severe-to-moderate neurological impairments, such as boccia players. Therefore, this study aims to: (i) design three sport-specific coordination tests for boccia players, evaluating their capability to discriminate between two sports classes (BC1 and BC2); (ii) to evaluate the relationship between generic and sport-specific coordination tests for a better understanding of whether they assess similar dimensions of impaired coordination; and (iii) to quantify how much coordination is impaired in boccia players compared to individuals without neurological impairments.

## Materials and Methods

### Participants

Seventy-three participants with CP (42 men and 31 women), from national (44%) and regional (56%) boccia competition levels, such as BC1 [*N* = 33; age = 34.01 ± 16.43 years; weight = 44.35 ± 13.88 kg; Gross Motor Functioning Classification Scale (GMFCS) scores = 3.89 ± 0.46] or BC2 [*N* = 40; age = 33.97 ± 14.29 years; weight = 50.44 ± 11.46 kg; GMFCS = 3.12 ± 1.04], were recruited to participate voluntarily in this study. All participants met the following inclusion criteria: (i) having a brain impairment from CP or a similar neurological condition; (ii) being classified as BC1 (spastic or athetoid quadriplegia or a mixture, including those with severe ataxia) or BC2 (spastic quadriplegia or with athetosis or ataxia) by BISFed (2); (iii) having had no surgeries or botulinum toxin injections in the 6 months prior to testing, which could impact on players’ motor function; and (iv) being able to follow the pertinent test instructions given by the researchers. The exclusion criteria were as follows: (i) athletes classified as BC3 (i.e., not able to grasp and release a boccia ball), BC4, or BC5 (i.e., non-central nervous system impairments) or (ii) players displaying intellectual impairments (21) (i.e., participants presenting limitation to understand the aims of the study or testing protocols). In addition, a group of 19 adults without any physical impairments was also included in the study (age = 27.89 ± 7.08 years; weight = 71.18 ± 11.55 kg) as the control group (CG). Ethics approval was obtained from the local University Ethics Committee (Ref. DPS-RVV-001-10). All participants provided their written informed consent prior to data collection.

### Procedure

This study was composed of two different data collection phases. During the first stage, a group of 45 participants with CP (BC1 = 23, BC2 = 22) and the CG (n = 19) performed four tests: two of them focused on assessing MD, and the other two were finger-tapping tests to assess ILC. In the second stage, a different group of 28 participants with CP (BC1 = 10, BC2 = 18) performed the same four tests as given above, plus two sport-specific tasks to assess ILC.

#### MD tests

The MD tests grouped together two tests that followed similar protocols in terms of grasping, transporting, and releasing an object. Both MD tests registered the number of objects (blocks or balls) that participants were able to handle and transport in 1 min.

##### Box and Block Test

This test was conducted according to the original instructions proposed by Mathiowetz and Volland (22). Participants used their throwing hand and performed two trials of 1 min, with 1 min of rest between the trials. Participants had 10 s of practice to familiarize themselves with the test. Excellent and high intraclass correlation (ICC) coefficients were demonstrated previously in similar samples of CP individuals (ICC = 0.97) and healthy adults (ICC = 0.85) (23). The outcome of the test was the number of blocks (11.1 ± 0.1 g, 25 mm in size) passed in the testing period of 1 min.

##### Box and Ball Test

**Figure 1:**
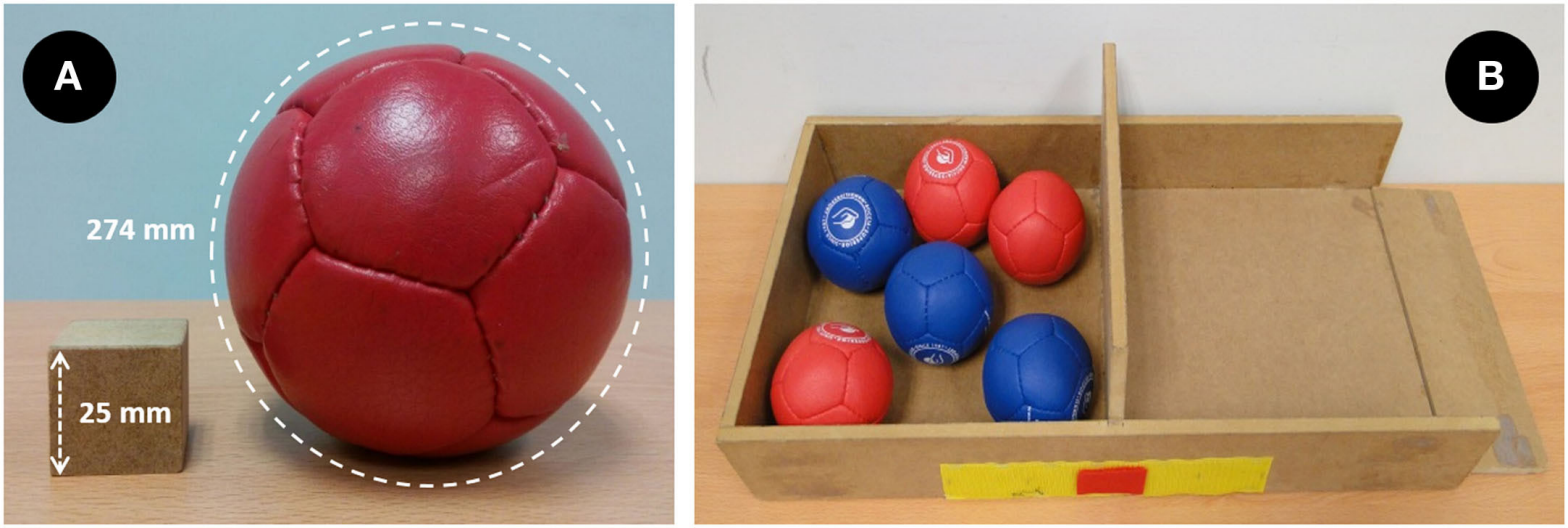
Block and boccia ball sizes **(A)** and Box and Ball setup **(B)**.

The BBLT followed the same procedure as the BBTs. The only difference was that the BBLT measured the number of boccia balls (Handi Life Sport, Skibby, Denmark: hard hardness, 278 g, 274 mm circumference, Figure 1A), instead of blocks, that an individual could transport in 1 min from one compartment to another. Due to the size of the compartments, only six balls could fit in a compartment at one time (Figure 1B). Two researchers were required, one at each side of the table. One researcher picked up the balls that had been left in the second compartment by participants and sent them (rolling across the table) to the second researcher, who refilled the first compartment when participants were releasing the ball. Reina et al. (23) reported excellent reliability for this adaptation of the BBT, both for participants with CP (ICC = 0.98) and adults without disabilities (ICC = 0.93).

#### Intralimb Coordination

The test battery to assess arm coordination grouped four different tapping tests, following similar protocols to those described by Connick et al. (19) and Deuble et al. (20), with good reliability. The three discrete ILC tests assessed movement time average (s) of the arm, while the continuous test assessed the number of taps (*n*) that each participant was able to make between plates during the testing period.

##### Discrete Horizontal Finger Tapping Test (DHFTT)

**Figure 2:**
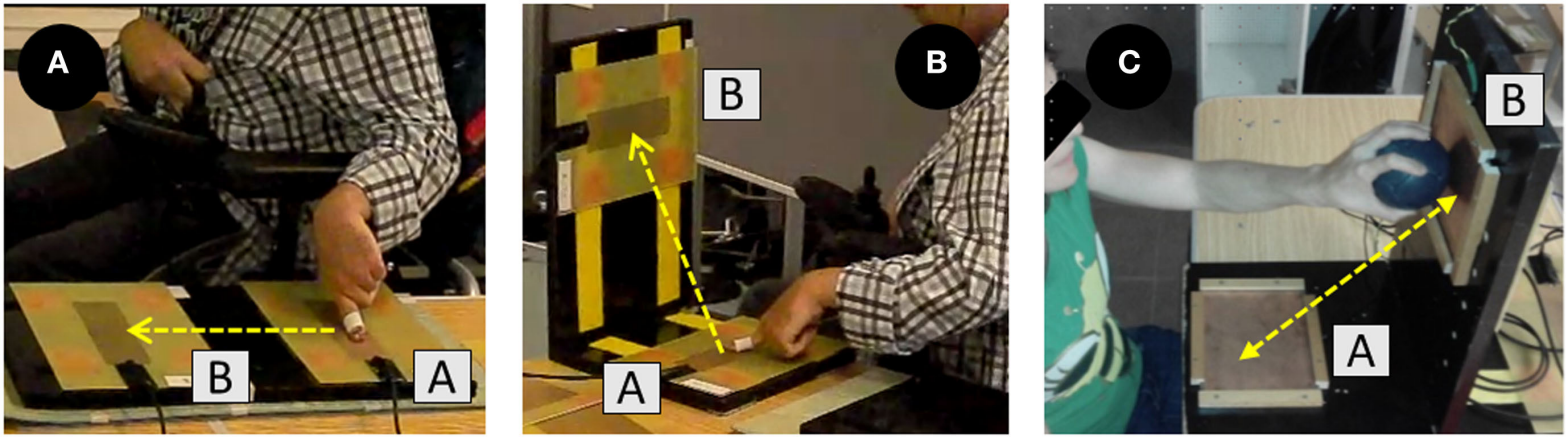
Discrete Horizontal Tapping Test **(A)**, Discrete Vertical Tapping Test **(B)**, and Discrete Vertical Tapping Test with Ball **(C)**.

Participants sat in their own wheelchairs and were placed parallel to a table, at 10 cm from the edge of the tapping plates. The table was adjusted so that the bottom of the table aligned with the players’ hips (greater trochanter), and the shoulders of the players’ throwing arms were aligned with the plate A (start position). Participants were asked to place their non-throwing arm across their chest and keep their throwing hand closed with the index finger extended (Figure 2A). However, due to some motor limitations (e.g., severe spasticity), not all participants were able to position their shoulders or fingers as requested, and these participants were thus allowed to place them in the most comfortable position for them as long as it did not interfere with the test execution. To complete the test, participants needed to complete 10 tapping cycles, reporting their performance as the average score of the 10 tapping cycles (s). A cycle was composed of the participants releasing from plate A to hit the plate B (finish position) as fast as possible. The plates were displaced horizontally, and the distance between both plates’ centers was 30 cm. The metal plates were 30 cm long by 20 cm wide. The target area, placed in the center of both plates, was 18 cm long by 5 cm wide. Any contact out of the target area was not registered. Once participants touched plate B, they had to return their finger to plate A. A period of at least 3 s had to pass between the trials, and participants were instructed to not move their finger until the researcher gave the start signal with the verbal command “Go!” The purpose of this test is to assess how fast, in seconds, an individual can move his or her finger from one plate to the other. Connick et al. (19) reported high-to-excellent intersession reliability for this test with young participants without disabilities (ICC = 0.85).

##### Discrete Vertical Finger Tapping Test (DVFTT)

The plates were arranged vertically in an “L” shape (90°), where plate A was kept on horizontal but plate B was placed on the vertical edge (Figure 2B). The distance between both plates’ centers was 30 cm, like in the DHFTT, and the two tests used the same protocol. The purpose of the DVFTT is also to assess how fast, in seconds, an individual can move his or her finger from one plate to another. Connick et al. (19) also reported high-to-excellent intersession reliability for this test (ICC = 0.92) in individuals without disabilities.

##### Discrete Vertical Tapping Test with Ball (DVTTB)

A new “L” shape structure and a new set of plates were laid out, using plates that worked through a spring system (i.e., the plates moved backwards when contact with the ball was made). Contact could be made at any point on the plates (14 cm × 17 cm). This test followed the same protocol as the DVFTT, in that participants used their throwing hand to complete 10 tapping cycles (i.e., contact with plate B). Participants sat in front of the table, with their throwing shoulder aligned with the center of plate A, 30 cm from the edge of the plate B. The purpose of this test is also to assess how fast, in seconds, an individual can move his or her finger from one plate to another. The intrasession reliability of this test was explored in this study (ICC = 0.87).

##### Continuous Vertical Tapping Test with Ball (CVTTB)

Participants performed a continuous movement consisting of grasping the ball and hitting plates A and B alternatively as fast as possible within 1 min. Ball contact could be made on any part of the plates. The start position in this test was outside of plate A, and data logging was activated upon first contact with the plate A after the signal “Go!” Participants practiced for 10 s to familiarize themselves with the motion before recording. The total number of times contact was made (i.e., touching both plates A and B) was recorded (Figure 2C). This test demonstrated high intrasession reliability in this study (ICC = 0.88).

### Data Acquisition

A video camera (Sony HDR-PJ410B) was placed on a tripod (Hama Star 63) in front of the participants to count the blocks and balls. In addition, a timekeeper (Casio HS-30W-1V) was used to control the testing time in both MD tests (BBT and BBLT). Regarding the ILC, to record the number of finger taps on the plates’ surfaces (DHFTT and DVFTT), the participants wore a metallic thimble. Each tap on the plate surface closed an electric loop, sending a signal that was registered with an A/D converter (USB-6001, National Instruments, Austin, TX, USA). For the tapping tests involving a boccia ball (DVTTB and CVTTB), two pressure plates were designed to register the ball contact in each movement. In this case, the pressure on the plates was the trigger that produced the electric impulse registered with the A/D converter mentioned previously. Data from the A/D converter were registered with a program developed within LabVIEW^®^ 2009 software (version 2.04, National Instruments, TX, USA).

### Statistical Analysis

The descriptive results are presented as the mean (M) and SDs. The normal distribution of the coordination tests results was tested using the Kolmogorov–Smirnov test with the Lilliefors correction, and a Levene’s test was conducted to check variances homogeneity. The coefficient of variation (CV, in %) was calculated within groups using the following formula (24): $\text{CV}=\left(\frac{\text{SD}}{\text{M}}\right)\times 100$. The interpretation of ICCs as reliability index, included in previous sections, was calculated according to Portney and Watkins (25): ICC values above 0.90 were considered excellent, values between 0.75 and 0.90 good, and values below 0.75 poor to moderate.

The relationships among different coordination tests performed by participants with CP were assessed using Pearson’s (parametric) and Spearman’s (non-parametric) product moment correlation (*r*). The following scale of magnitudes was used to evaluate correlation coefficients: <0.1, trivial; 0.1–0.3, small; <0.3–0.5, moderate; <0.5–0.7, large; <0.7–0.9, very large; and <0.9–1.0, almost perfect (26).

A one-way analysis of variance (ANOVA) with a least significant difference *post hoc* comparison (Tukey’s correction) was used to examine the mean differences between the CP subgroups (i.e., BC1 and BC2) and the CG in those tests where parametric techniques were pertinent; while Kruskal–Wallis analysis was conducted in those tests where non-parametric techniques were required. In addition, a paired-samples t-test (parametric) and Wilcoxon test (non-parametric) was conducted to evaluate other performance differences within groups: (1) MD tests (BBT vs. BBLT); (2) discrete finger-tapping tests (horizontal vs. vertical); and (3) discrete vertical-tapping tests (without vs. with ball). The practical significance was assessed by calculating Cohen’s effect size (ES). ESs of above 0.8, between 0.8 and 0.5, between 0.5 and 0.2, and lower than 0.2 were considered large, moderate, small, and trivial, respectively (27). A correction by Hedges’ *g* index (*d_g_*) was used for the comparisons within groups (28).

All the data analyses were performed using the Statistical Package for Social Sciences (SPSS Inc., version 24.0, Chicago, IL, USA). The statistical significance was set at *p* < 0.05.

## Results

The Levene’s analysis (*W*) revealed that the three discrete tapping tests (DVTTB, *W* = 0.03; DVFTT, *W* = 0.04; and DHFTT, *W* = 0.01) did not have variance homogeneity, and non-parametric techniques have been used for data analysis (Spearman’s correlation, Kruskal–Wallis, and Wilcoxon analyses). On the other hand, the analyses for the continuous tests (BBT, *W* = 0.12; BBLT, *W* = 0.82; and CVTTB, *W* = 0.34) were conducted with parametric techniques (Pearson’s correlation, one-way ANOVA with honestly significant difference (HSD) Tukey’s *post hoc*, and paired-samples *t*-test analyses).

Correlation analyses of the tests were conducted (Table 1) to determine whether the new sport-specific tests assessed the same coordination dimensions as the generic tests. A very large correlation was obtained with the two MD tests (*r* = 0.80; *p* < 0.01), but the discrete tapping tests received a moderate-to-very large negative significant correlation (−0.30 < *r* < −0.75; 0.05 > *p* < 0.01). A large positive correlation was discovered with the continuous tapping test with a ball (0.59 < *r* < 0.66; 0.05 > *p* < 0.01), and a moderate negative significant correlation was obtained with the two tapping tests (continuous vs. discrete) that required grasping a ball (*r* = −0.47; *p* < 0.05). Finally, a large-to-very large positive significant correlation between the discrete coordination tests was shown (0.62 < *r* < 0.86; *p* < 0.01).

**Table 1:** Pearson’s (a) and Spearman’s (b) product–moment correlations between coordination tests in participants with CP.

	BBT	BBLT	CVTTB	DVTTB	DVFTT	DHFTT
BBT (N blocks)^a^	–	0.802**	0.656**	−0.753**	−0.294*	−0.447**
BBLT (N balls)^a^			0.593*	−0.482**	−0.302*	−0.434**
CVTTB (N contacts)^a^				−0.468*	−0.519**	−0.390**
DVTTB (s)^b^					0.860**	0.775**
DVFTT (s)^b^						0.623**
DHFTT (s)^b^						–

*BBT, Box and Block Test; BBLT, Box and Ball Test; CVTTB, Continuous Vertical Tapping Test with Ball; DVTTB, Discrete Vertical Tapping Test with Ball; DVFTT, Discrete Vertical Finger Tapping Test; DHFTT, Discrete Horizontal Finger Tapping Test; CP, cerebral palsy*.

*^a^Pearson’s correlation*.

*^b^Spearman’s correlation*.

*p < 0.05; **p < 0.01.

Table 2 shows the performance scores of the two boccia player groups and the CG. Differences between the groups (*p* < 0.001) were obtained in all the coordination tests. The HSD between groups was calculated with a Tukey’s *post hoc* (continuous tests) and Kruskal–Wallis (discrete tests) analyses, and the difference between the CG with respect to both CP groups was obtained (*p* < 0.01; 1.60 < *d* < 10.28, large). Comparing the two groups of boccia players, significant differences were also obtained in the two MD tests (*p* < 0.01; 0.93 < *d* < 1.13, large) and in the continuous tapping test that required grasping a ball (*p* < 0.01; *d* = 1.12, large). However, no significant differences were obtained between the two groups of participants with CP in the three discrete finger- and ball-tapping tests (0.41 < *d* < 0.55, small-to-moderate).

**Table 2:** Performance scores by participants with CP (BC1 and BC2) and the CG.

Test	Group	*N*	M ± SD	CV (%)	*F*(df)	*p*	*d*^(Tukey’s^ *^post hoc^*^differences)^		
							BC1 − BC2	BC1 − CG	BC2 − CG
BBT (N blocks)	BC1	33	19.29 ± 6.86	35.56	710.41 (2, 89)	<0.001	−0.93**	−10.28**	−7.21**
	BC2	40	27.12 ± 9.83	36.25					
	GC	19	86.16 ± 6.12	7.10					
BBLT (N balls)	BC1	33	23.46 ± 11.36	48.42	601.37 (2, 89)	<0.001	−1.13**	−7.93**	−6.98**
	BC2	40	35.81 ± 10.51	29.35					
	GC	19	96.78 ± 6.48	6.70					
CVTTB (N contacts)	BC1	10	28.60 ± 13.46	47.06	205.40 (2, 44)	<0.001	−1.12**	−8.46**	−6.40**
	BC2	18	44.90 ± 15.69	34.92					
	GC	19	129.33 ± 10.11	7.82					

**Test**	**Group**	***N***	**M ± SD**	**CV (%)**	**χ^2^ (df)**	***p***	***d*****^(Kruskal–Wallis pair comparisons)^**		
							**BC1 − BC2**	**BC1 − CG**	**BC2 − CG**

DVTTB (s)	BC1	10	1.21 ± 0.86	71.04	53.59 (2, 44)	<0.001	0.50	1.60**	2.00**
	BC2	18	0.87 ± 0.44	50.88					
	GC	19	0.24 ± 0.07	30.35					
DVFTT (s)	BC1	33	0.98 ± 0.53	53.81	47.60 (2, 89)	<0.001	0.41	1.90**	2.62**
	BC2	40	0.80 ± 0.28	35.31					
	GC	19	0.27 ± 0.06	24.44					
DHFTT (s)	BC1	33	1.05 ± 0.64	61.62	34.45 (2, 89)	<0.001	0.55	1.76**	3.07**
	BC2	40	0.78 ±0.24	30.95					
	GC	19	0.24 ± 0.06	23.43					

*BBT, Box and Block Test; BBLT, Box and Ball Test; CVTTB, Continuous Vertical Tapping Test with Ball; DVTTB, Discrete Vertical Tapping Test with Ball; DVTTF, Discrete Vertical Finger Tapping Test; DHTTF, Discrete Horizontal Finger Tapping Test; BC1/BC2, participants with severe neurological impairments, Boccia classes; CG, control group; df, degrees of freedom; d, Effect size*.

**p < 0.01.

Table 3 shows the comparisons within groups for the MD tests (BBT vs. BBLT), discrete tapping tests without a ball (DHFTT vs. DVFTT), and the discrete vertical-tapping test (with or without a ball). Comparing the scores of the two MD tests showed that all three groups transported a higher number of balls than blocks (*p* < 0.001). In addition, comparing the performance between the two discrete vertical-tapping tests (with and without a boccia ball), the two CP groups (BC1 and BC2) showed a slower performance when test required to grasp the ball (−2.02 < *z* < −2.53; *p* < 0.05; −0.24 < *d* < −0.42, small).

**Table 3:** Within-groups pair comparisons in MD and ILC coordination tests.

	BBT vs. BBLT			DHFTT vs. DVFTT			DVFTT vs. DVTTB		
	*t*	*p*	*d_g_*	*z*	*p*	*d_g_*	*z*	*p*	*d_g_*
BC1	−3.94	<0.001	−0.59	−0.85	0.398	0.11	−2.53	0.011	−0.42
BC2	−7.97	<0.001	−0.87	−0.44	0.657	−0.08	−2.02	0.043	−0.24
CG	−10.72	<0.001	−1.66	−0.11	0.913	−0.48	−0.72	0.470	0.48

*BBT, Box and Block Test; BBLT, Box and Ball Test; DVTTB, Discrete Vertical Tapping Test with Ball; DVTTF, Discrete Vertical Finger Tapping Test; DHTTF, Discrete Horizontal Finger Tapping Test; BC1/BC2, participants with severe neurological impairments, Boccia classes; CG, control group; d_g_, effect size with Hedges’s correction*.

## Discussion

This study aimed to develop sport-specific coordination tests for boccia players and compare their concurrent validity with other generic coordination tests in order to (i) discriminate between BC1 and BC2 sport classes and (ii) quantify the level of impaired coordination that these players have compared to individuals without disabilities.

Significant correlations were obtained among all the coordination tests: a very large correlation for the MD tests and a moderate-to-large correlation for the ILC tests. These results support the hypothesis that the new sport-specific coordination tests assess similar dimensions of impaired coordination to generic tests in individuals with CP who are eligible to play boccia. The different magnitudes of the correlations across the coordination tests may indicate that the test protocol and its requirements constrain the participants’ performance.

### MD Tests

Our results demonstrated that boccia players displayed MD limitations in comparison to the CG. The MD tests were able to discriminate between individuals in different sport classes (BC1 vs. BC2), presenting the BC1 players as having worse performance scores. These results correspond to those of Golubović et al. (7), who carried out the BBT with children with different degrees of CP and showed that children with higher levels of impairment (e.g., quadriplegia) transferred smaller numbers of blocks compared to less affected children.

When comparing performance levels in the MD tests (see Table 2), one can observe that all the groups obtained better performance scores in the BBLT than in the BBT (i.e., they transported a higher number of balls than blocks). It is also plausible to think that the new BBLT has a better discriminant capacity (i.e., slightly higher ES), as some specific features of the new sport-specific test might have influenced participants’ proficiency. For example, the size and the shape of the object were shown to influence the type of grip used, as well as the kinematic features that the children with CP used to transport any object (29, 30). Extrapolating this information to the MD tests of this study, it was observed that the boccia balls were larger and featured a curved design, which fit better into the participants’ hands. This could have led to a more precise coordination of the hand muscles and, therefore, a more powerful grip (31). Wright et al. (30) found that children with hemiplegic CP needed more hand motor adjustments, as they demonstrated slowness in, for example, flexing and overextending their fingers when the manipulative task became more difficult (e.g., manipulation of a cylindrical vs. a triangular object). A second aspect to take into account is the gripped objects’ frictional surfaces. Some materials facilitate the coupling between a hand or finger and an object. In our case, the blocks used in the BBT, which had a wooden surface, easily slipped from participants’ fingers and thus required more precise fingertip coordination to perform the test (32). On the other hand, the leather surface of the boccia balls improved players’ handgrip function, increasing the hand–object frictional coupling (33). Hence, the BBLT demands a more realistic grasp ability from the players due to the specificity of the object (size and material).

It is important to mention that all the players in this study were able to grasp a ball and throw it with direction and intention (2), which are the main criteria for eligibility as a hand player in boccia (i.e., BC1 or BC2).

### Intralimb Coordination

Discrete tasks are understood as actions that require a single response with a clear beginning and ending, while continuous tasks are understood as reciprocal actions with no recognizable beginning and end that flow on for a specific period (34). Furthermore, each type of task presents different kinematic features (35) that should be considered during arm coordination assessments.

Our results demonstrated that boccia players tend to display ILC limitations in comparison to individuals without CP. The CG demonstrated shorter movement times in the discrete tapping tests (DVFTT, DHFTT, and DVTTB) and a higher rate (i.e., number of taps) in the continuous tapping test (CVTTB). On the other hand, boccia players performed worse due to muscle weakness, impaired voluntary muscle activation, and problems regarding muscle coactivation, as it has been found in similar studies on children with CP (36, 37).

Comparing the sport classes, the BC1 players performed worse overall in all the coordination tests (Table 2). However, only the continuous tapping test with a boccia ball was able to discriminate between sport classes. Given the motor characteristics of a throw, discrete tasks were expected to be more sensitive in discriminating between sport classes, but no statistical significance was found. However, the ESs of the three discrete tasks (DVFTT, DHFTT, and DVTTB) were moderate, indicating some practical differences. These results can be explained by a potential ceiling effect of the discrete tasks due to their simplicity. Discrete tests usually require less motor control, fewer adjustments, and less movement planning to perform more controlled and accurate movements (34). In these tasks, boccia players were able to self-regulate according to their impairment level, choosing the most optimal pacing to perform the tasks as fast as possible. This lack of a challenge can explain why the discrete tasks were not effective in discriminating between sport classes. Lajoie et al. (38) found similar results working with older adults, not finding significant differences when participants performed simple discrete conditions compared to other tasks that required a greater cognitive engagement (i.e., more difficult tasks).

The continuous tapping test seemed to present a major challenge for the boccia players, requiring more motor control, movement planning, and sensory information processing to perform it (39). Altered muscle tone and muscle coactivation is a typical feature in individuals with hypertonia, especially in antigravity muscles (40). This impairment could cause worse performance, decreasing players’ movement efficiency (41), reducing movements’ velocity (42) and hindering continuous elbow flexion–extension movements. On the other hand, players with severe dyskinetic or ataxic profiles may display uncontrollable muscle contractions, tending to activate the antagonist muscles before the agonist (43). Therefore, when they tried to perform the continuous test with abnormal force, pacing, and accuracy, they required more time to complete the coordination task. Similar results were found in young individuals with dystonic CP, who showed abnormal timing and coordination during functional arm movements affecting the upper extremities (39). Thus, it was expected that the most severe players would end with worse timing scores, as BC1 players showed.

Another aspect to consider in the tapping tests to assess ILC was the use of an implement (i.e., the boccia ball). Therapists have repeatedly shown that the use of added-purpose activities enhances motor performance in comparison to isolated and repetitive exercises (44). Thus, handling an implement may increase the task difficulty, demanding multiple actions at the same time: holding and transporting the ball to the target, as this study shows in the discrete vertical tapping tests (higher scores holding the ball). The challenge becomes even more complex when the individual with CP displays hand and arm coordination problems, supported by the large ES obtained in the CVTTB. Therefore, grabbing a ball while performing reaching movements might cause, due to diminished selective motor control, an increase in hand and arm muscle tone (45).

These results must be interpreted with caution because BC1 players have the largest CV in all the coordination tests, indicating that this group displays the most heterogeneous proficiency. In any case, continuous tasks seem to be more relevant for assessing impaired coordination in individuals with eligible neurological impairments for boccia. However, future studies should address to what extent impaired coordination determines boccia performance.

### Limitations

Some limitations should be mentioned. First, some results in this study should be interpreted with caution, and further studies to assess validity are needed in order to implement these tests in sport classification (e.g., intersession test validity designs). Second, although the observed power in the continuous tests was adequate (i.e., size of the sample), a larger and a more homogeneous sample should be required for the discrete tapping tests, where non-parametric techniques were used for data analysis.

## Conclusion

Neurological impairments may impact hand and arm structures, such as muscles, joints, and bones, limiting one’s ability to perform manual activities (e.g., grasping, releasing, or manipulating objects) crucial for performing daily-life activities or taking part in sport like Boccia. Currently, the Boccia classification system does not have sport-specific instruments to assess hand–arm coordination in boccia players. The test battery presented in this study seems to be a feasible means of assessing impaired coordination in adults with moderate-to-severe CP.

To discriminate between different levels of coordination impairment it is important to consider certain testing features (e.g., object size and shape or type and direction of the movement), especially when studying the feasibility of new measuring instruments, both in para-sport or clinical settings. This study shows how continuous movements (for MD and ILC) seem to have a higher capacity to distinguish between individuals with and without CP and among different levels of neurological impairments. On the other hand, the complexity of the task should also be considered. For example, this study revealed that repetitive movements (i.e., as required in the discrete tapping tests) may not be complex enough to discriminate among individuals with severe-to-moderate CP. Therefore, the authors would suggest increasing the difficulty adding a task constraint, such as holding a ball.

## Ethics Statement

This study was carried out in accordance with the recommendations of Miguel Hernández University Ethics Committee (Ref. DPS-RVV-001-10) with written informed consent from all subjects. All subjects gave written informed consent in accordance with the Declaration of Helsinki. The protocol was approved by the Miguel Hernández University Ethics Committee (Ref. DPS-RVV-001-10).

## Author Contributions

AR, RS, and RR contributed in the conception and design of the study; all the authors took part in the acquisition, analysis, and interpretation of data; contributed drafting the article and critically revising the article for important intellectual content; and AR, DB, and RR approved the last version to be published.

## Conflict of Interest Statement

The authors declare that the research was conducted in the absence of any commercial or financial relationships that could be construed as a potential conflict of interest.
